# High-Quality Draft Genome Sequence of *Leucobacter* sp. Strain G161, a Distinct and Effective Chromium Reducer

**DOI:** 10.1128/genomeA.01760-15

**Published:** 2016-02-18

**Authors:** Shimei Ge, Wenjing Ai, Xinjiao Dong

**Affiliations:** College of Life and Environmental Science, Wenzhou University, Wenzhou, China

## Abstract

Here, we report the genome sequence for *Leucobacter* sp. strain G161 due to its distinct and effective hexavalent chromium reduction under aerobic growth conditions, followed by facultative anaerobic incubation. The draft genome sequence of *Leucobacter* sp. G161 comprises 3,554,188 bp, with an average G+C content of 65.3%, exhibiting 3,341 protein-coding genes and 55 predicted RNA genes.

## GENOME ANNOUNCEMENT

The genus *Leucobacter* was first described as comprising yellow-pigmented, Gram-positive, aerobic, non-spore-forming, irregular rod-shaped bacteria in the phylum *Actinobacteria*, belonging to the class with high G+C content (1). This genus currently includes 15 species and two subspecies, which have been found in a variety of environments. The reported 9 strains were chromium resistant, including *L. chromiiresistens* (2), *L. alluvii*, *L. luti* (3), *L. chironomi* (4, 5), *L. aridicollis*, *L. chromiireducens* (6), *L. salsicius* (7), and two subspecies, *L. chromiireducens* subsp. *chromiireducens* subsp. nov. and *L. chromiireducens* subsp. *solipictus* subsp. nov. (8).

Our research focus, *Leucobacter* sp. strain G161, was isolated from chromate-contaminated soil, which was collected from a tannery factory in Wenzhou, China (9). This isolate exhibited chromate resistance and hexavalent chromium reduction. It is worth mentioning about the distinct chromium-reducing conditions for *Leucobacter* sp. G161. The organism showed no growth under anaerobic conditions but reduced Cr(VI) under all conditions. Furthermore, *Leucobacter* sp. G161 demonstrated the most effective Cr(VI) reduction during aerobic growth, followed by facultative anaerobic incubation (9). Although there has been genomic information available for *Leucobacter* spp. with chromium resistance (4, 10–13), the chromium-reducing ability of *Leucobacter* sp. G161 was unique and particular, which may have important implications for the bioremediation of facultative chromium-contaminated circumstances. The in-depth genome sequencing will give more insights into the molecular mechanisms of the distinctive Cr(VI) reduction and provide some basis for the bioremediation of chromium contamination by using this microbe or the chromium reduction mechanism.

The draft genome of strain G161 was sequenced via an Illumina MiSeq system using the 2 × 301-bp paired-end read sequencing strategy. The genome coverage was approximately 618×. The sequence assembly was performed using Velvet 1.2.09 (best k-mer, 81 bp), and finally resulted in 109 contigs (>200 bp), with a total length of 3,554,188 bp (*N*_50_, 94,114 bp) and a G+C content of 65.3%.

The annotation of the genome was performed using the RAST annotation server (14) and NCBI Prokaryotic Genome Annotation Pipeline (PGAP) (http://www.ncbi.nlm.nih.gov/genome/annotation_prok/). The *Leucobacter* sp. G161 genome contains 3,257 predicted coding sequences and 55 predicted RNAs. Nucleotide BLAST at EzBioCloud (http://www.ezbiocloud.net/) and phylogenetic tree analysis based on the full-length 16S RNA gene sequence were used to attempt to identify the species of strain G161. The results showed that *Leucobacter* sp. G161 is most closely related to *L. komagatae* IFO 15245, with a similarity of 98.9%, and it had much more divergence with other *Leucobacter* species. Therefore, we are unable to assign a species name to this isolate, and it may be a novel candidate species.

### Nucleotide sequence accession numbers.

The whole-genome shotgun project of *Leucobacter* sp. G161 has been deposited at DDBJ/EMBL/GenBank under the accession no. LOHP00000000. The version described in this paper is version LOHP01000000.
